# Adolescent urology: A challenge for adult urologists

**DOI:** 10.4103/0970-1591.35049

**Published:** 2007

**Authors:** C. R. J. Woodhouse

**Affiliations:** Department of Urology, University College, London, UK

**Keywords:** Adolescence, urology

## Abstract

The medical care of adolescents has become a growth area in many disciplines. There are two major aspects. Firstly, adolescents have specific medical and emotional needs which are not fulfilled either by paediatric or by adult specialists. Secondly, some childhood problems, particularly the congenital deformities, have no equivalent in adult life and so lifelong care is mandatory. Renal damage, especially dysplasia and scarring, leads to a substantial risk of early onset hypertension and, occasionally, to renal failure. Bladder outlet obstruction in utero, such as from a posterior urethral valve, causes irreversible changes to the wall that will act adversely on the kidneys in adolescence or early adulthood. The incidence of renal failure in early adulthood is about 36%. Bladder reconstruction with bowel has been very beneficial in preventing renal failure and improving continence. Life long follow-up is needed because of the high incidence of complications. These include stones, hyperchloraemic acidosis, perforation, anastomotic stenosis and, possibly, cancer. Patients have a normal expectation of sexuality and fertility. Their desires cannot always be achieved but they require considerable emotional and surgical support.

Adolescent medicine is a growing area in many specialties. Table 1 shows the exponential rise in publications in the 20^th^ century from a Pubmed search. I think the figures can be treated with some skepticism as key words were not used for most of the period and casual examination of several cited papers reveals no connection with adolescents! Nonetheless, the graph for urology in the second half of the century clearly illustrates the increasing interest.

**Table 1:** Graphs to show the number of publications by year listed in Pubmed for articles on adolescents (blue) and adolescent urology (pink) in the 20^th^ Century. Note that it uses a logarithmic scale

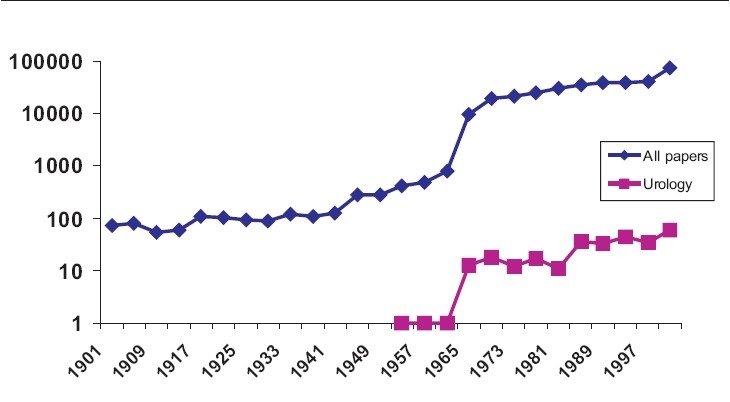

In University College Hospital there now are departments for almost all disciplines [Table 2]. Throughout the world, increased survival of children with major congenital anomalies and acquired childhood diseases is producing a generation of individuals who have all of the problems of adolescence combined with the continuing effects of their illness. There is a major problem for society and for doctors in arranging their care.

**Table 2:** Adolescent departments in University College Hospital, London

With adult equivalent	Without adult equivalent
Asthma	Cardiology
Diabetes	Oncology
Rheumatology	Orthopaedics
In patient care	Neurology
	Endocrinology
	Gynaecology
	Disorders of Sexual Development
	Urology

Conditions in the left column have an equivalent in adult life. Those in the right column usually have no such equivalent (e.g.: there is no adult equivalent of exstrophy while a juvenile with diabetes will become an adult with diabetes)

Most conditions are rare in the population as a whole. Particularly in those conditions which have no equivalent in adult life, it is inappropriate to pass care from pediatric specialists to the local adult specialist. This is particularly true in urology. An adult urologist cannot be expected to look after patients born with, for example, exstrophy or posterior urethral valves, when he is likely to have seen only one in his entire life.

Unfortunately, adolescents are perceived to be rude, difficult, aggressive and, above all perhaps, poor. Their care as a specialty is, therefore, very unappealing. In state-run systems, there is some prospect of proper funding but there is still the problem of setting up super-specialist clinics to deal with small numbers of patients. In the case of conditions with no adult equivalent, the care will have to be lifelong.

In largely privately funded systems, such as the USA, it is almost impossible to provide universal care, a situation highlighted in the New York Times in 2007 (Sunday New York Times, Supplement, 15^th^ April 2007).

This article will do no more than outline the spectrum of problems encountered in the long-term follow-up of children born with major genitourinary anomalies. It might be considered an appeal to pediatric and adult urologists in India to arrange continuing care for a most deserving group of patients.

## THE KIDNEYS

Damage to the kidneys occurs in fetal life in many of the congenital urological anomalies. Although the structural anomaly, such as a posterior urethral valve, may be corrected just after birth (or even in utero) the kidneys may never recover so that hypertension and renal failure develop in later life. Renal follow-up is an integral part of adolescent care. Blood pressure measurement, tests for urinary protein and for serum creatinine must be a part of the annual review.

**Figure 1:**
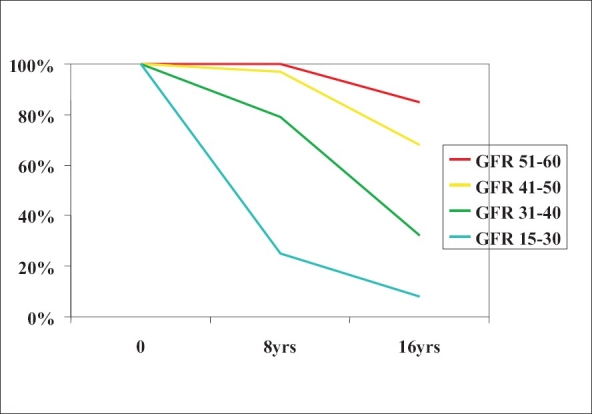
Percentage of patients surviving without dialysis/transplantation by initial GFR corrected to 1.73m^2^. Time zero was at entry into adolescent followup around puberty[1]

Patients who reach adolescence with a glomerular filtration rate (GFR) of less than 40 ml/min/1.73 m^2,^ have a substantial chance of developing end stage renal failure within 16 years [Figure 1].[1] The downward progress can be slowed with rigorous control of proteinuria and blood pressure with angiotensin conversion enzyme inhibitors (ACEI).

The cause is probably hyperperfusion injury of surviving nephrons, but a watch must be kept for continuing damage to the kidneys by the bladder, especially in boys born with posterior urethral valves. It should be remembered that if the bladder has killed two native kidneys, it may do the same to a transplant. The bladder must be made safe before a transplant can be contemplated.[2]

**Figure 2:**
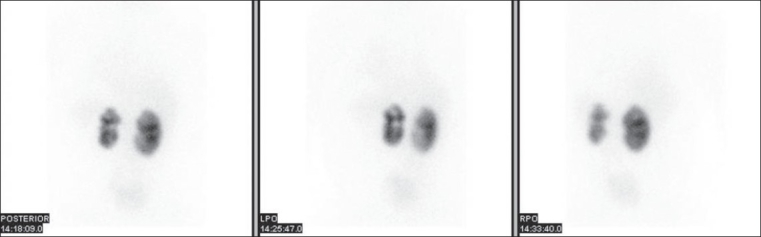
Tc-99m DMSA scan showing bilateral cortical scarring. The changes are more profound on the left side compared to the right. The left kidney contributes 37% to total renal function; right 63% (Courtesy Dr J. Bomanji)

Damaged kidneys cause hypertension. Even a small scar may be incriminated. If scars are suspected, a DMSA scan is essential [Figure 2].

The risk of hypertension has been best documented in patients with reflux nephropathy. In assessing risk, however, it is essential to remember that normal blood pressure increases with age. The traditional cutoff for normality of 140/90 mmHg is not appropriate for adolescents and age-adjusted norms must be used.

The range given in the literature for the incidence of hypertension is very wide, partly from the definition of age used and partly from the inclusion criteria of the study. The risk certainly increases with age. It seems likely that a patient with unilateral nephropathy at 18 years of age has a 12% risk of hypertension.[3] At the top end, a 27-year-old with bilateral nephropathy may have a risk as high as 44%.[4]

## BLADDER FUNCTION

Classically, the bladder is described as an organ for the storage and evacuation of urine. However, at all ages, but particularly in the young, the function of the bladder may have a profound effect on renal function. The monitoring of the one cannot be separated from that of the other.

The major problems are seen in boys born with posterior urethral valves and all born with neural tube defects.

It is essential to understand that the main long-term consequences of posterior urethral valves (PUV) occur because of damage to the bladder by obstructed voiding *in utero* and in the neonatal period before the valve is resected. They consist of loss of compliance, detrusor instability and reduced functional capacity. In adulthood, the pattern may change to one of chronic retention, often with high pressure.

Although not seen in all PUV boys, the potential for such damage is always present and may not be manifest until adolescence or early adult life. The symptoms will be familiar to all urologists as frequency, urgency and incontinence. What may not be recognized is the relentless effect on the kidney.

The progressive nature of renal failure in PUV boys has been recognized for many years. In 1988 the incidence of renal failure was 23% at 10 yo, 30% at 16 yo and 43% at 30 yo.[5] A normal serum creatinine at the beginning of puberty does not mean that it will be normal at the end. In spite of a greater understanding of the pathophysiology of the valve bladder, these figures have not greatly improved (18%, 32% and 36% respectively in 2005).[6]

Apart from the renal damage caused by the unrelieved obstruction before the valve resection, there is ongoing damage from the poorly compliant bladder. There is a suggestion from recent work that renal failure may be prevented by aggressive bladder management with anticholinergics, self-catheterization and clam cystoplasty.[7]

Unfortunately, time is not kind to any aspect of myelodysplasia, especially the bladder. Detrusor function changes rapidly in the first two to three years of life. It stabilizes in childhood, but tends to deteriorate further in puberty. The late deterioration is a consequence of increasing outflow resistance, changing habits of life and, in some, the effects of the tethered cord.

Early institution of active bladder management can reduce the long-term damage, help bladder function and reduce the risk of renal failure, at least in childhood. The burden of this system on the family is considerable as it involves intermittent catheterization of the infant and administration of anti-muscarinic drugs. However, by 7 yo 92% of children have normal kidneys and 77% are continent.[8]

Artificial urinary sphincters can be used in children with myelomeningocoele. About two-thirds of patients will be made continent but the half life of sphincter survival is about eight years.[9]

Spontaneous development of continence at puberty, especially in boys, usually heralds disaster. The leak-point pressure has increased and the kidneys are at risk. At least a half of such patients will develop new renal dilatation and, if untreated, will go on to renal failure.[10]

Bladder augmentation is a useful option in myelodysplasia. It exposes the child to the same risks as in other patients. Stones, perforation and changes in bowel habit are particularly important in this group.

The effect of a tethered cord is much disputed with some authorities claiming that the entity does not even exist! However, change in voiding or defecation patterns in an adolescent is an indication for early neurological re-evaluation. Release of a tether may stabilize function, but may bring improvement in 60%.[11]

## RESERVOIRS AND AUGMENTED BLADDERS

All urinary reservoirs made of intestine have the same basic long-term problems. Hyperchloremic acidosis occurs in up to 14% of patients though many more may have a metabolic acidosis with respiratory compensation. Anemia occurs in 8%.[12] Vitamin B_12_ deficiency is a risk after five years, especially if terminal ileum is used.[13] Fortunately, they seem not to cause renal deterioration except in the presence of some complication such as ureteric obstruction.[14]

Reservoir stones occur in 12-15% of patients. Risk factors include infection, need for self-catheterization and retained mucus. Recent work has suggested that there may be metabolic abnormalities in some patients with enterocystoplasty that predispose to stone formation. The most important are low urinary citrate and high pH.[15]

Reservoir perforation is a lethal complication which is, fortunately, rare. Delayed diagnosis and inadequate treatment may lead to the death of the patient. The diagnosis is best made on clinical grounds and aspiration of abdominal fluid collections identified by ultrasound. Unless there is improvement within a few hours on antibiotics and intravenous fluids, laparotomy is essential.[16]

Cancer is an important long-term risk. A distinction must be made between neoplasms of a retained bladder, those of an augmenting intestinal segment and those of an anastomosis between the urothelium and intestine.

**Figure 3:**
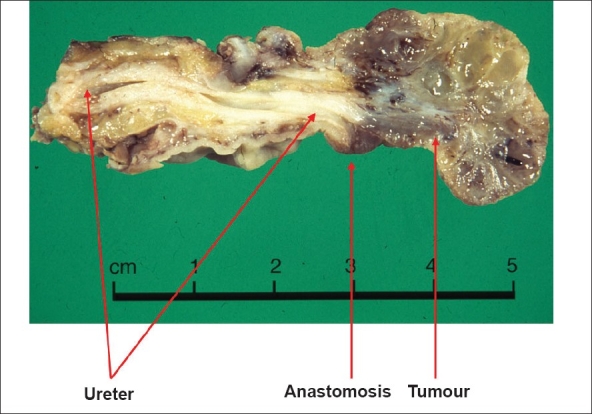
Photograph of a specimen of ureter with an anastomotic tumor removed from a patient with ureterosigmoidostomy

The anastomotic neoplasm classically is seen in patients with ureterosigmoidostomy [Figure 3]. The incidence of all neoplasms is 19% at 20 years of follow-up and 33% at 35 years. How many of these are malignant will depend on the vigilance of follow-up, benign lesions probably taking a mean of five years to become malignant. In our own series, where most cases are found by surveillance flexible sigmoidoscopy, malignant tumors were found in 7% at 20 years and 9% at 35 years.[17]

It now is becoming apparent that in reservoirs with feces excluded, there is still a risk of neoplasia. In a recent review, 49 cases of reservoir malignancies were identified in the literature.[18]

More worrying are figures from a long-term follow-up of 260 children from a single center who had a bladder augmentation. At a minimum of 10 years, three had developed transitional cell carcinoma of the bladder. This incidence of 1.6% is far higher than would be expected in such young individuals.[19]

Patients with an augmented bladder with urethral access should have a flexible cystoscopy annually from the 10th year. Where access to any reservoir is only through a Mitrofanoff, the problem is more difficult. A standard flexible cystoscope may be too large to pass. A smaller one may have too small a field of view definitely to *exclude* a tumor.

Manipulation of the instrument in the Mitrofanoff channel may damage continence. It may be that diagnosis will have to rely on regular ultrasound, augmented if necessary with a rigid cystoscopy through a suprapubic puncture.

## SEXUAL FUNCTION AND FERTILITY

It is self-evident that adolescents are very interested in sex. With a modicum of maturity, they become interested in fertility or least with the means of preventing it. The burden of a major congenital anomaly does not lessen these interests. It is amazing how much sexual enjoyment can be had even with very abnormal genitalia. The duty of the adolescent urologist is to inform, encourage and, sometimes, to operate to allow the potential to be fulfilled.

**Figure 4:**
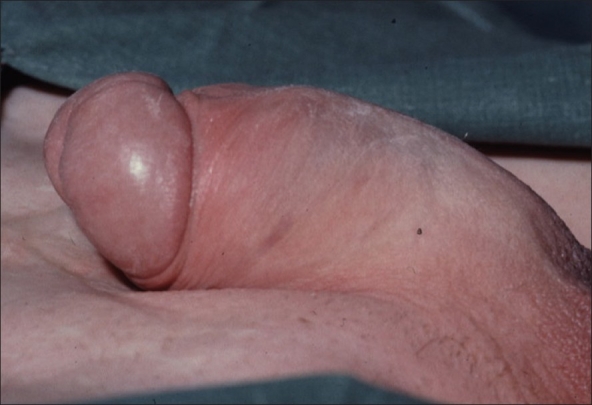
Clinical photograph of the erect penis in a man with exstrophy illustrating tight dorsal chordee

Such problems are well illustrated in those born with exstrophy. The penis is short but of normal caliber. An intrinsic part of the anomaly is tight dorsal chordee. If not corrected in infancy, the deformity will prevent penetrative sexual intercourse [Figure 4]. In adults, the deformity is investigated by artificial erection, infusing both corpora with saline. Minor defects may be corrected by a ventral Nesbit's procedure, severe ones by a Cantwell-Ransley operation.

**Figure 5:**
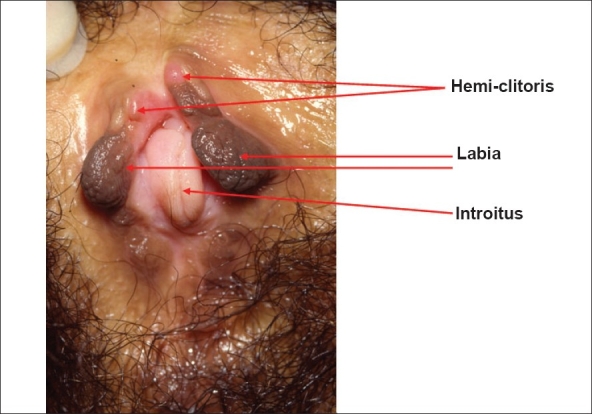
Clinical photograph of the perineum of a woman with exstrophy. There is no urethra as she has a suprapubic diversion

In females the vagina is short and lies in a more horizontal plane than normal. The labia are anterior and often on the anterior abdominal wall. The introitus is narrow. The clitoris is bifid [Figure 5]. Although the position of the labia cannot be changed, the whole abnormality can be disguised and made completely functional. Patients are normally fertile.[20]

Uterine prolapse occurs in up to 50% of females and is unrelated to pregnancy.[20]

There are so many operations for the repair of hypospadias that the non-specialist may despair of ever understanding even the basic techniques. By adult life, however, most patients have had a satisfactory repair, at least by the standards of the surgeon. Surgeons reviewing their own results give glowing reports such as a straight erection and normal urine stream in 100%. With objective review, the results may be poor in up to 50%.[21]

There is no consensus on the effect of hypospadias on sexual development, if only because today's adults were reconstructed using techniques that have been superseded.

**Figure 6:**
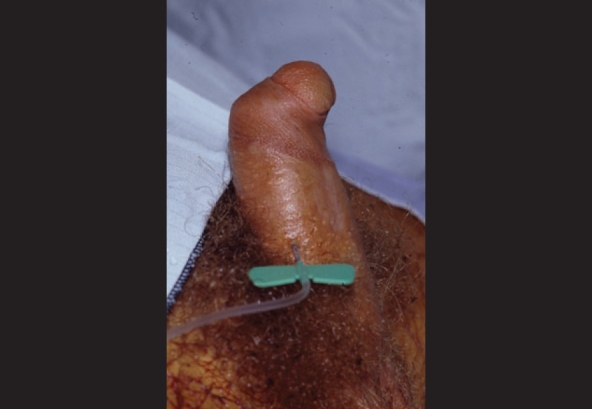
Operative photograph of a man with uncorrected hypospadias and chordee

If there are gross persistent anomalies, especially chordee, intercourse may be impossible [Figure 6]. With lesser anomalies, particularly of a cosmetic variety, sexual debut and intercourse may be nearly normal. When compared to boys operated for hernia or for phimosis, there are no differences in the development of standard sexual milestones.[22] Minor seminal anomalies may be seen in up to 50%, but there does not seem to be an increased incidence of infertility.

In adolescents with spina bifida there are sexual problems related to the neurological defect as well as difficulties that result from their dependent status. The physical aspects of sexual function that depend on the brain are generally intact, while those which depend on the spinal cord will be damaged in line with the neurological level.

**Figure 7:**
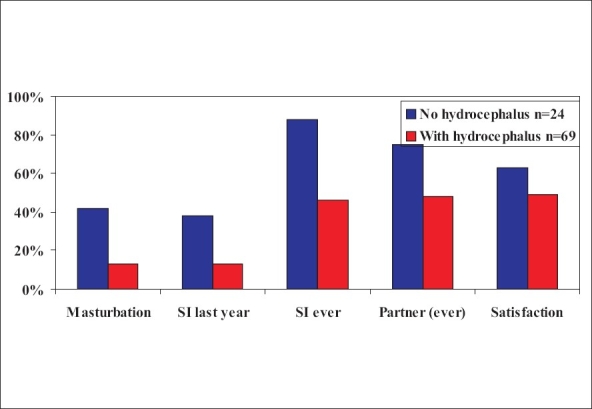
Histogram showing sexual activity in women with spina bifida[23]

In females sexual function is closely related to spinal level and bladder continence. Most females with levels below L2 are normal as are most with urinary continence. Only about 20% of those with higher levels or with urinary incontinence have normal sexual function. A recent study from the Netherlands has shown that, in adolescents, sexual activity is less common than might have been suspected, especially in those with hydrocephalus [Figure 7].[23]

All males with intact sacral reflexes and urinary continence are potent. With absent sacral reflexes, 64% with levels below D10 and 14% with levels above D10 are potent. There is doubt about the true sexual nature of such erections.[24]

**Figure 8:**
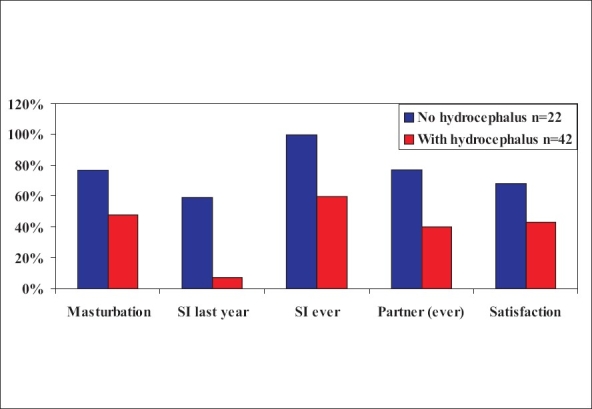
Histogram showing sexual activity in men with spina bifida[23]

Again, the male patients in the Dutch study had less than expected sexual activity, especially if they had hydrocephalus [Figure 8].[23]

Impotence responds to conventional management such as intracorporeal injection.[25] Sildenafil may be used with appropriate dose reduction. In the only trial to date in this group, dose escalation was used with patients as their own controls. Eighty per cent of men responded to a dose of 50mg. Although one patient subsequently responded to 100mg, it was recommended that such a high dose should not be used in spina bifida. Five of the 11 responders in the series were wheelchair bound.[26,27] In view of the possibility that impotence is associated with azoospermia, the prospects for fertility may not be improved.

In later adult life, two-thirds form steady sexual relationships, regardless of the degree of handicap or continence. Pregnancies are difficult with high rates of urinary infection and deterioration in bladder function. The risk of neural tube defect in the offspring is 1:23 overall (1:50 for sons and 1:13 for daughters); the risk is significantly reduced with folic acid supplement for three months before conception and in the first trimester of the pregnancy.[28]

Many anomalies also affect fertility and pregnancy. Advances in reproductive technology have improved matters very considerably, but also bring problems of stress and expense.

Poor male fertility is seen in exstrophy, prune belly syndrome, renal failure, disorders of sexual development (intersex) and spina bifida. Boys born with a unilateral undescended testis (UDT) probably have normal fertility. Those with bilateral UDT successfully operated in childhood have about a 50% chance of fathering a child.

Poor female fertility is seen in chronic renal failure, disorders of sexual development (including congenital adrenal hyperplasia) and anomalies of uterine development.

There is a specific problem in confirming pregnancy in women whose urine is stored in an intestinal reservoir: the standard HCG antibody (blue line) urine test is commonly positive in the absence of pregnancy.

Pregnancy itself has particular problems in women with spina bifida, enterocystoplasty, re-implanted ureters and exstrophy. However, almost all resolve after delivery. Women should always be advised of such problems but few are deterred from having a baby as a result.

In women with a GFR below 50 ml/min/1.73 m^2^ and a diastolic blood pressure (untreated) above 90mmHg, there are very significant risks of irreversible renal deterioration. Between a third and two-thirds will precipitate into end stage renal failure, about 20% will have worse hypertension and 20% worse proteinuria after delivery.[29]
